# Physiological Plasticity to Water Flow Habitat in the Damselfish, *Acanthochromis polyacanthus*: Linking Phenotype to Performance

**DOI:** 10.1371/journal.pone.0121983

**Published:** 2015-03-25

**Authors:** Sandra A. Binning, Albert F. H. Ros, David Nusbaumer, Dominique G. Roche

**Affiliations:** 1 Division of Evolution, Ecology and Genetics, Research School of Biology, The Australian National University, Canberra, Australia; 2 ARC Centre of Excellence for Coral Reef Studies, The Australian National University, Canberra, Australia; 3 Eco-Éthologie, Institut de Biologie, Université de Neuchâtel, Neuchâtel, Switzerland; Sonoma State University, UNITED STATES

## Abstract

The relationships among animal form, function and performance are complex, and vary across environments. Therefore, it can be difficult to identify morphological and/or physiological traits responsible for enhancing performance in a given habitat. In fishes, differences in swimming performance across water flow gradients are related to morphological variation among and within species. However, physiological traits related to performance have been less well studied. We experimentally reared juvenile damselfish, *Acanthochromis polyacanthus*, under different water flow regimes to test 1) whether aspects of swimming physiology and morphology show plastic responses to water flow, 2) whether trait divergence correlates with swimming performance and 3) whether flow environment relates to performance differences observed in wild fish. We found that maximum metabolic rate, aerobic scope and blood haematocrit were higher in wave-reared fish compared to fish reared in low water flow. However, pectoral fin shape, which tends to correlate with sustained swimming performance, did not differ between rearing treatments or collection sites. Maximum metabolic rate was the best overall predictor of individual swimming performance; fin shape and fish total length were 3.3 and 3.7 times less likely than maximum metabolic rate to explain differences in critical swimming speed. Performance differences induced in fish reared in different flow environments were less pronounced than in wild fish but similar in direction. Our results suggest that exposure to water motion induces plastic physiological changes which enhance swimming performance in *A*. *polyacanthus*. Thus, functional relationships between fish morphology and performance across flow habitats should also consider differences in physiology.

## Introduction

What drives performance differences among individuals and species? This fundamental question has fascinated researchers for decades [1–6]. Although many studies have suggested that diversity in morphological and/or physiological traits contributes to inter- and intra-specific differences in performance, the relative importance of these traits in driving observed performance differences as well as the role of the environment in shaping phenotypic divergence remain important areas of investigation [3,7,8]. Understanding the functional relationships between phenotype and performance can also provide a strong framework for predicting species distributions and patterns of selection and evolution across habitats [7].

In aquatic systems, water motion is a critical physical factor that influences the distribution and phenotype of species based on their capacity to cope with high flow [7,9,10]. Functional relationships between morphology and water flow have been repeatedly documented in inter- and intra-specific studies of freshwater and marine organisms across multiple spatial scales [11–19]. In fishes that use their pectoral fins to generate thrust (median-paired fin (MPF) labriform swimmers), the distribution of species and individuals across water flow gradients is strongly related to the biomechanical relationship between pectoral fin shape and swimming performance [20,21]: tapered fins tend to be faster steady swimmers (i.e. straight-line swimming at a constant velocity), whereas rounded fins tend to be optimized for manoeuvring in low-flow habitats [20,22–24]. This functional relationship appears useful for predicting patterns of species assemblages and ecology based on easily measured morphological traits [6]. However, as many authors have noted, the actual links between organismal design, function and performance are complex, and represent the integration of numerous traits under the influence of multiple selective pressures which differ among environments [3,7,25–27]. As a result, the direct influence of a single morphological trait, such as fin shape, on swimming performance may be weak. In addition, physiological traits such as increased aerobic capacity and blood oxygen carrying capacity are more complex to measure in swimming fishes, but may well contribute to observed performance differences.

We explored the relationships among organismal form, performance and the environment by studying intraspecific trait variation related to water flow habitat in a labriform-swimming coral reef fish, *Acanthochromis polyacanthus*, a planktivorous damselfish (Pomacentridae) in the Coral Sea and Great Barrier Reef (GBR). Recent studies suggest that individuals of this species vary greatly in terms of their swimming performance (prolonged swimming speed) depending on water flow habitat across both large and small spatial scales [24,28,29]. These performance differences have been dually attributed to divergence in morphological (fin shape) and physiological (metabolic rate) traits among populations [28,29]. However, the degree to which each of these traits contributes to differences in performance, and whether trait divergence is the result of genetic adaptation or a plastic response to flow environment remains unknown.

*Acanthochromis polyacanthus* lacks a pelagic larval stage, and broods can remain with their parents for several months [30,31]. As a result, population divergence occurs over spatial scales as little as 3 km when sites are separated by deep-water channels (> 10 m depth). Despite high levels of genetic relatedness within populations, there is no evidence of inbreeding in this species on the GBR [32]. The unusual life-history of this species as well as variation in traits related to swimming ability across flow habitats provide a foundation for exploring trait polymorphisms related to performance and the influence of the environment in driving divergence across small spatial scales [32,33].

We reared juvenile *A*. *polyacanthus* in a split-brood experiment to test 1) whether exposure to water flow habitats during development elicits plastic changes in physiological and/or morphological traits related to swimming, and 2) whether these changes enhance the swimming performance of individuals. We then 3) compared swimming performance, metabolic rate and fin aspect ratio obtained from our rearing experiments with data from wild adult fish collected from the same windward and leeward sites to evaluate whether performance differences observed in reared fish help explain the phenotypic differences observed in wild individuals.

## Materials and Methods

### Ethics Statement

Research was conducted under permits from the Great Barrier Reef Marine Park Authority (G11/34413.1) with approval from the ANU Animal Experimentation Ethics Committee (B.EEG.03.10).

### Fish sampling and experimental design

We collected fish from 16 broods at Lizard Island, northern Great Barrier Reef, Australia (14° 40’ S; 145° 28’ E). Eight broods were on the predominantly windward (wave exposed) and eight on the leeward (sheltered) side of the island. Patterns of water motion (wave height and water flow velocity) differ from six up to 15-fold between these sites during windy conditions [34,35]: average water flows are approximately 37 cm s^-1^ versus 9 cm s^-1^ at windward and leeward sites respectively. Broods were selected based on their developmental stage (stage 2–3, 4 to 6 weeks old) [31], distance apart (> 30 m between broods), and brood size (> 30 fry). Developmental stage was chosen to maximize the chance of survivorship in the aquarium setting and enable broods to be uniquely identified using Visible Implant *Elastomer* Tags (VIE tags, North-West Marine Technologies). Only broods with two parents and no other similar-stage families within 10 m were selected to increase the likelihood that all collected individuals from a brood were full-siblings. We collected fish using 10% Aqui-S solution and hand nets, and transported them in plastic bags to the aquarium facilities at Lizard Island Research Station within 1h of capture. Each brood was housed in separate aquaria (40.0W× 29.0L× 18.0H cm) with a flow-through water system directly from the reef. Each aquarium was provided with two cylindrical PVC shelters (9.0L×5.5H cm). At this stage, fish were monitored every 2h during daylight to ensure individuals were in good health. There were no fish deaths during this time. Fish were fed approximately 0.25g NRD 2/4 pellets (Primo aquaculture) per tank each morning and at dusk. After three days, we measured and injected 24 individuals from each brood (384 fish total, *L*_T_ = 3.3 ± 0.1 cm; mass = 0.5 ± 0.1 g; means ± s.e.m.) with VIE tags using 1cc gauge syringes to uniquely colour-code siblings. Fish were given two days to recover and then assigned to treatment tanks. Left-over fish not used in the experiments were returned with their siblings to their site of capture, and where possible, their parents.

Four identical outdoor rectangular aquaria (50.0W×100.0L× 35.0H cm, 175 litres) were fitted with two Vortec MP40 (Ecotech Marine) propeller pumps (one on each end, lengthwise across the tank) programmed to operate alternately and create unsteady flow conditions simulating a wavy, reef crest-like habitat (period = 2 sec, wave amplitude = 6 cm). Pumps were initially fitted with protective cages (13.0W×16.0L× 12.0H cm) covered with 10 mm stretch monofilament mesh to prevent access by small fish. These cages were removed after four months. Flow velocity in the tanks was estimated by video-tracking passive particles at various depths and locations within each tank to ensure similar flow conditions across all four tanks. Videos were filmed with a digital camera (Exilim EX-FH100, Casio, USA) fixed vertically over the tanks and analysed using the MtrackJ plugin in the ImageJ v. 1.43 software. Flow ranged between 0.0–27.5 cm s^-1^ (19.5 ± 0.15 cm s^-1^, mean ± s.d.) when cages were present, and 0–48.7 cm s^-1^ (35.2 ± 3.4 cm s^-1^, mean ± s.d.) when cages were removed. Four additional aquaria were assigned to the calm treatment and supplied with bubblers (water flow < 4.0 cm s^-1^) to simulate calm lagoonal conditions with low water flow. Mesh covers prevented predation by birds. Water in the tanks was fed via a flow-through system which draws water directly from the reef, and the tanks experienced natural light and temperature regimes throughout the experiment (February- October 2012). All tanks contained three cylindrical PVC pipe shelters (large; 15.0L×11.0H, medium; 9.0L×5.5H, small; 7.0L×11.0H) affixed to the bottom with silicone to provide shelter and the opportunity for fish to flow-refuge, which is a commonly observed behaviour in wild coral reef fishes [36].

Three individuals from each brood were randomly assigned to each tank (48 individuals per 175 L tank, 3.6 L of water per fish). Fish in each tank were fed 0.75g NRD 2/4 pellets (Primo Aquaculture, Australia) each morning and dusk for six weeks, then switched to 0.25g NRD 2/4 pellets and 0.75g NRD 5/8 pellets twice a day. After four months, feeding rations were changed to 0.5g NRD 5/8 pellets and 0.5g NRD G12 pellets per tank twice a day. After seven months, feeding rations were changed to 1.0g NRD G12 pellets per tank twice a day for the remaining 6 weeks of the experiment. Fish were retagged after six weeks and four months to ensure the VIE tags remained visible. Organic material deposited in the tanks was siphoned out weekly. Organic material deposited in the tanks was siphoned out weekly. Tanks were monitored three times daily (during morning and evening feeds, and once at mid-day), and any sick or dead individuals noticed during these checks were immediately removed. Sick individuals were identified by lesions on the skin surface and fins, difficulty swimming, maintaining stability and/or controlling buoyancy. Sick individuals were removed from the tanks, anaesthetised in a 10% Aqui-S solution, and then euthanized in an ice-slurry. An infection in one of the unsteady flow tanks caused the mortality of all 48 individuals after 3.5 months, and therefore all measurements, experiments and analyses were performed on fish from the remaining seven tanks (N = 336 fish). Mortality in the remaining tanks during the course of the experiment was low (< 5%). All healthy individuals were released at their site of capture after the completion of the experiments.

### Fin morphology

To estimate pectoral fin shape, fish were sedated in a water bath containing 10% Aqui-S solution, and their extended fin was photographed on gridded waterproof paper with a digital camera (Panasonic Lumix DX3) [37]. We measured the length of the leading edge and total fin area using ImageJ software (V 1.43). Pectoral fin aspect ratio (AR) was then calculated as the length of the leading edge squared divided by the total fin area. Only undamaged fins were analysed. We photographed pectoral fins twice during the rearing experiment, once after four months and once prior to the end of the experiment (eight months). The four month fin measure was taken to assess the consistency of our fin measures through time, and to assess whether ontogenetic changes in fin shape occur, which may have affected the interpretation of our results. Only one fish per brood from each tank was randomly selected and measured after four months to minimize stress and potential damage to the fins. In total, we measured 107 individuals after 4 months (total length *L*_T_ = 7.8 ± 0.0 cm; fish wet mass M = 3.9 ± 0.0 g; mean ± s.e.m.) and 186 individuals after 8 months (*L*_T_ = 10.1 ± 0.1 cm; M = 17.7 ± 0.2 g, mean ± s.e.m). Fin AR measurements were averaged for siblings in the same experimental tank for the eight month measurement (N = 101 in the analysis)

### Swimming performance and metabolic rate measurements

We measured critical swimming speed (*U*_crit_), and oxygen consumption rates (*Ṁ*O_2_: mg O_2_ h^-1^) in September and October 2012 after fish had spent a minimum of seven months in the experimental treatments. Due to time and equipment constraints, we were unable to run a balanced design with tank as a factor in the analysis. To control for tank effects, we selected fish from tanks at random until one individual from each of the 16 broods had been tested from each treatment (32 fish total, *L*_T_ = 10.4 ± 0.1 cm; M = 19.0 ± 0.4 g, mean ± s.e.m.). Fish were fasted for 24h prior to the swimming trials to standardize a post-absorptive state that maximizes energy availability for swimming. We obtained length measures for individual live fish before swimming trials by holding each fish in a plastic bag half-filled with water to minimize stress due to air exposure and measuring *L*_T_, body width (BW) and body depth (BD) with handheld callipers. Wet mass was measured directly on a scale and involved minimal air exposure (<10s). These measures were inputted into the respirometry software and used to calculate the flow velocity in fish total lengths per second (*L*_T_ s^-1^). Fish were placed into an aerated bucket to recover for 30 minutes before being transferred to the swim tunnel using silicone hand-nets. Care was taken to minimize air exposure during this time, and most transfers took approximately 1–2 seconds.

We measured *Ṁ*O_2_ as a function of swimming speed (*U*) following a *U*_crit_ protocol [38]. Swimming trials were carried out in a 4.8 L custom-built Steffensen-type swim tunnel respirometer using intermittent-flow respirometry [39,40]. The swim chamber dimensions were 30.0 L × 7.0 W × 7.0 H cm. Temperature was maintained at a constant 26 ± 0.1°C (actual variation) using a TMP-REG temperature analyser and regulator system (Loligo Systems, Denmark). Oxygen levels in the respirometer were recorded using a fibre optic oxygen meter (Presense Fibox 3) online feed into the AutoResp 1 Software (Loligo Systems, Denmark). The flow in the working section of the respirometer was calibrated using a digital TAD W30 flow-meter (Höntzsch, Germany). Solid blocking effects of the fish in the working section of the respirometer were corrected by the respirometry software (AutoResp, Loligo Systems) following [41]. The cross-sectional area of fish was always less than 9% of the chamber cross-sectional area corresponding to approximately 4% greater effective water velocity around fish compared to the water velocity in the empty swim chamber [42].

We used ten minute loops with a 240 s flush, 60 s wait and 300 s measure cycle. Once an individual’s length and mass measures were inputted into the respirometry software, three loops were run without a fish to measure initial background rates of respiration due to bacterial load in the test chamber. The fish was then placed in the respirometer and left to acclimate for five to eight hours at a swimming speed of 1.0 *L*_T_ s^-1^ until its oxygen consumption rate had reached a steady state (did not increase or decrease be more than 5% for over 1 h) and the fish had settled into a continuous swimming rhythm [28,43–45]. This speed corresponded to the lowest water flow necessary to ensure constant swimming and minimize spontaneous activity in *A*. *polyacanthus* (Binning, pers. obs.). We measured oxygen consumption rate at 1.0 *L*_T_ s^-1^ by averaging the three *Ṁ*O_2_ measurements immediately prior to the onset of the first trial [28,44]. Flow speed was then incrementally increased by 0.5 *L*_T_ s^-1^ every three 10-minute loops (i.e. every 30 minutes) for the duration of the experiment. As *A*. *polyacanthus* approaches *U*_crit_, it transitions from steady pectoral fin swimming first to caudal-assisted and then to unsteady burst-and-coast swimming, which marks the transition from purely aerobic to mixed aerobic/anaerobic powered swimming for labriform swimmers [28,46]. When an individual approached fatigue and began resting on the back of the grate, the experimenter would gently tap first the back then the sides of the chamber to encourage swimming [47]. The trial stopped when the fish could no longer swim unassisted and was forced to rest on the back of the flow chamber (*U*_crit_) for five or more seconds despite experimenter tapping [43,44,47]. The time and water flow speed was recorded and the water flow was reduced back to *L*_T_ s^-1^ for at least 10 minutes to allow partial recovery from anaerobic burst-and-coast swimming. The fish was then removed from the test chamber and transferred to a separate holding tank. Three additional cycles were run without a fish to measure background *Ṁ*O_2_ in the empty chamber. Background oxygen consumption rates were determined from the slope of the linear regression between initial and final background oxygen consumption rates, and were subtracted from each *Ṁ*O_2_ estimate. The respirometer was drained and rinsed in freshwater every four fish to ensure that background consumption rates due to bacteria did not exceed 10% of the oxygen consumption rates of the fish at 1.0 *L*_T_ s^-1^. Fin AR was measured on fish one day following their swim in the respirometer following [37] and methods described above. All healthy fish were released at their site of capture within one week following their swim.

We calculated a fish’s critical swimming speed (*U*_crit_) following the equation in [39]:

Ucrit=U+Uix(t/ti)

where *U* is the penultimate swimming speed before the fish fatigued and stopped swimming; *U*_*i*_ is the swimming speed at which the fish was unable to continue swimming (i.e. swimming speed at increment *i*); *t* is the length of time the fish swam at the final swimming speed where fatigue occurred; *t*_*i*_ is the amount of time fish were swam at each speed interval in the trial (30 min).

We used a hydrodynamics-based power function to describe the relationship between *Ṁ*O_2_ and swimming speed (*U*) for each fish [44,48]:

$$\dot{\text{M}}{\text{O}}_{2}=\text{a}+\text{b}U^{c}$$

where *a* is *Ṁ*O_2_ at zero speed (*Ṁ*O_2, min_), which is an estimate of standard metabolic rate (SMR). *Ṁ*O_2, max_ or maximum metabolic rate (MMR) was estimated at a fish’s maximum swimming speed during prolonged swimming [44]. We calculated the aerobic scope (AS) as MMR minus SMR [49]. We also calculated factorial aerobic scope as the ratio between MMR and SMR [49]; this approach led to qualitatively similar results as those obtained with AS, therefore we only present AS.

### Blood parameters

In October 2012, we sampled blood in the laboratory facilities at LIRS from one randomly selected individual per brood for each treatment (32 fish total, *L*_T_ = 10.1 ± 0.8 cm; M = 18.2 ± 0.4 g, mean ± s.e.m.). Individuals were captured with a rubber mesh hand net, and immediately placed into a container containing 10% Aqui-S solution until equilibrium was lost (< 20 sec). A small blood sample (apx. 0.1 ul) was drawn from the caudal vasculature using a 25 gauge needle in a 1 ml syringe. Fish were then placed in a bucket of aerated seawater to recover. Blood was immediately transferred to micro-haematocrit capillary tubes and centrifuged in a fixed speed haematocrit centrifuge (10 000 rpm) for 5 min. The proportion haematocrit (Hct) was calculated as the ratio of red blood cells to total blood volume. Short-term changes in Hct levels caused by red blood cell swelling and splenic release can be induced by stress in fish [50]. Therefore, we took all efforts to minimize the time taken from capture to sampling (< 3 min per fish).

We also tested for differences in blood cortisol levels between the two water flow treatments groups. Cortisol has previously been used to assess both background stress levels as well as responses to acute stressors in coral reef fishes [51]. We used a species-independent commercial cortisol enzyme immunoassay kit (DetectX, Arbor Assays, Ann Arbor, Michigan USA). To avoid matrix effects, 10ul of plasma was extracted twice with 3ml of diethyl ether (recovers 95% of steroids). Diethyl ether was evaporated in a speedvac and the non-polar fraction was resuspended in 1ml of assay buffer. Immunoassays were carried out following the manual of the kit. Intra-assay precision was 10.6%CV. Cross reactivities reported by the kit are 1.2% for cortisone and <0.1% for progesterone.

### Statistical analyses

All analyses were performed in R v3.0.0 [52]. The assumptions of the models were assessed with diagnostic plots (plots of residuals vs. fitted values and qqplots of residuals) and transformations were used where necessary. We used general linear mixed models (LMMs; lme function in R) to compare MMR, aerobic scope (AS), and fin shape (AR) between collection sites (leeward or windward; fixed factor) and flow treatments (unsteady or calm; fixed factor) while controlling for brood identity (random factor) and fish size (covariate; fish mass for MMR and AS; fish *L*_T_ for fin AR). We calculated the marginal R^2^ (variance explained by the fixed factors; R^2^_GLMM(m)_) and conditional R^2^ (variance explained by the fixed and random factors;; R^2^_GLMM(c)_) following [53]. LMMs were also used to compare Hct (logit transformed [54]) and cortisol level (log_10_ transformed) between collection sites and water flow treatments (fixed factors), controlling for brood identity (random factor). Since Hct does not tend to vary with fish size, we did not include a covariate in this analysis [55].

We used model selection followed by model averaging to determine which morpho-physiological traits and environmental variables provided the best predictors of swimming performance in *A*. *polyacanthus*. We included fish *L*_T_, fin AR, MMR, collection site and rearing treatment as potential predictors of swimming performance (*U*_crit_), controlling for fish brood (included as a random factor). Fish mass and *L*_T_ were tightly correlated in our fish (*P* < 0.001, R^2^ = 0.62). We used the information theoretic approach for model selection and to assess model performance. We used LMMs to produce candidate models that examined the effects of the predictor variables on *U*_crit_. Prior to this analysis, we reduced the number of candidate models in our analysis by excluding two-way interactions that had no apparent effect on the response variable as determined from graphical examination of all biologically-meaningful two-way interactions [56]. We included all single terms, and allowed interactions between treatment and total length, treatment and MMR and treatment and AR. To select the best subset of models, we used Akaike’s Information Criterion modified for small sample sizes (AICc). For all candidate models that have substantial support as explanations of swimming performance (all models with normalized Akaike weights, *w*_*im*_, within 10% of the maximum weight), we performed model averaging and calculated the model-averaged estimates of parameters (β). To determine the reliability of the predictor estimates from averaging, we calculated the weighted unconditional standard error (SE) with its associated confidence intervals (95% CI) and the normalized Akaike weight for each predictor (w_*ip*_). Data associated with this manuscript are publically archived in the data repository figshare (http://dx.doi.org/10.6084/m9.figshare.923561) [57].

## Results

**Fig 1:**
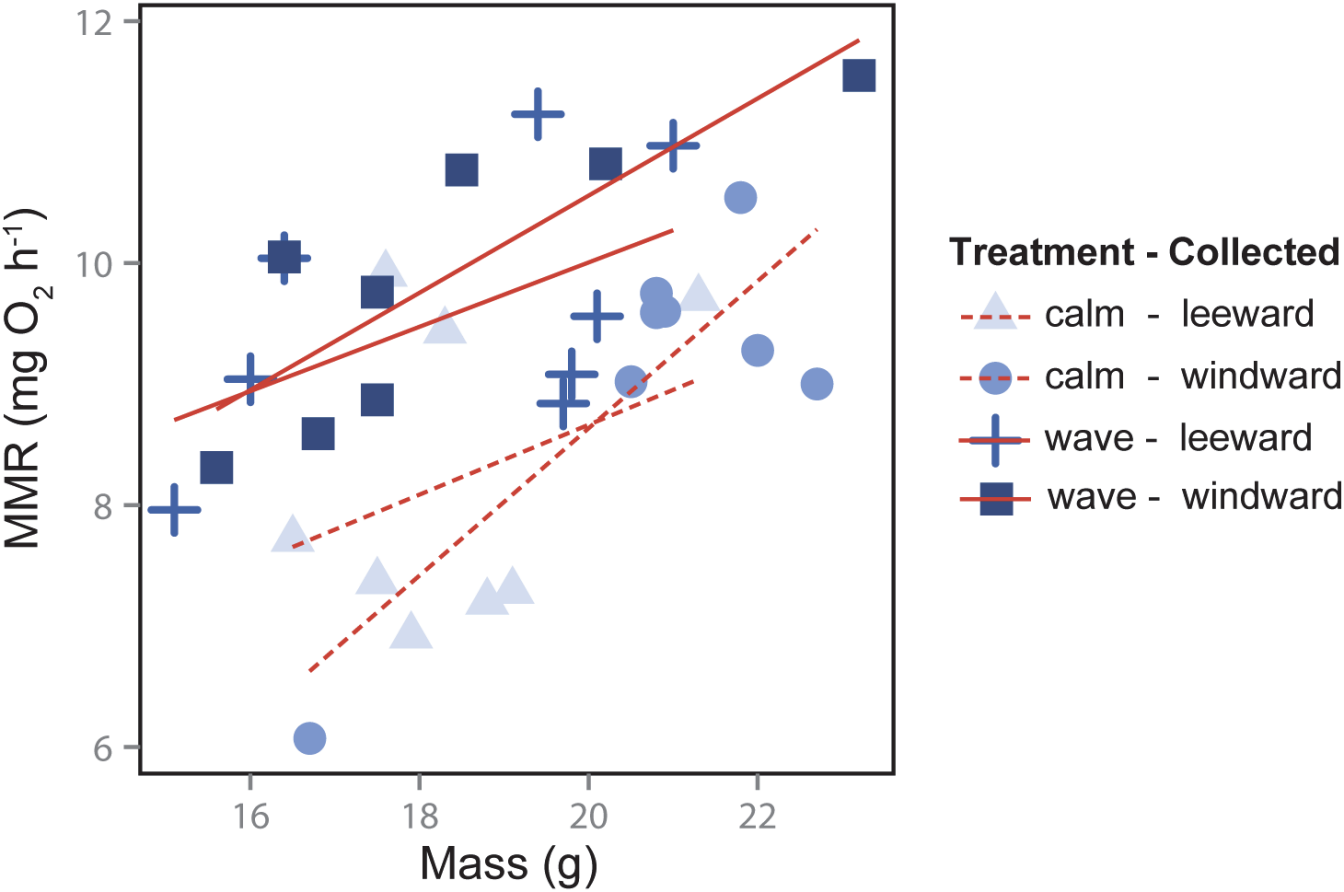
Maximum metabolic rate in relation to fish mass in *Acanthochromis polyacanthus*. Individuals from different collection sites (leeward vs. windward) and rearing treatments (wave vs. calm) are indicates with symbols. The solid and dashed lines represent the best fit linear regressions for wave and calm treatments, respectively.

Absolute MMR increased with fish mass (Fig. 1). When controlling for mass, there was a significant effect of rearing treatment on absolute MMR: wave-reared fish had higher MMR (relative and absolute) than calm-reared individuals (Tables 1, 2). This effect was independent of where fish were collected (no treatment by collection site interaction, no significant effect of collection site on MMR; Fig. 1, Tables 1, 2). The same differences were observed with AS, a measure of aerobic capacity (Tables 1, 2).

**Table 1:** Physiological and morphological traits in *Acanthochromis polyacanthus* from sites around Lizard Island.

**Birth site**	**Treatment**	M	*L*_T_	4 month AR	8 month AR	*U*crit	MMR	AS	Hct
		(g)	(cm)			*(L*_T_ s^-1^)	(mg O2 kg^-1^ h^-1^)	(mg O_2_ kg^-1^ h^-1^)	
**N**	-	8	8	27	25	8	8	8	8
**Leeward**	Calm	18.4 ± 0.5	10.6 ± 0.1	1.26 ± 0.08	1.28 ± 0.01	3.56 ± 0.06	446.83 ± 23.66	293.92 ± 19.93	0.32 ± 0.04
**Leeward**	Water flow	18.4 ± 0.8	10.2 ± 0.2	1.24 ± 0.06	1.23 ± 0.01	4.15 ± 0.05	523.56 ± 21.03	402.05 ± 23.60	0.38 ± 0.02
**Windward**	Calm	20.8 ± 0.6	10.8 ± 0.1	1.25 ± 0.09	1.27 ± 0.01	3.66 ± 0.09	436.73 ± 14.38	285.61 ± 11.36	0.31 ± 0.02
**Windward**	Water flow	18.2 ± 0.9	10.1 ± 0.1	1.30 ± 0.09	1.23 ± 0.01	4.16 ± 0.06	542.06 ± 14.13	393.16 ± 16.12	0.43 ± 0.04

Values are means ± s.e.m. Fish mass (M), total length (*L*_T_), mean pectoral fin aspect ratio (AR; measured after four and eight months), relative critical swimming speed (*U*_crit_), relative maximum metabolic rate (MMR), aerobic scope (AS) and proportion of blood hematocrit (Hct), number of fish measured per site and treatment (N).

**Table 2:** Analysis of covariance table.

		df	F-ratio	P-value
**MMR**	(Intercept)	1, 14	2121.7	<0.001
	**Mass**	**1, 11**	**10.14**	**0.009**
	Collected	1, 14	0.73	0.406
	**Treatment**	**1, 11**	**30.74**	**< 0.001**
	Mass X Collected	1, 11	0.58	0.46
	Mass X Treatment	1, 11	0.04	0.84
	Collected X Treatment	1, 11	1.21	0.30
R^2^_LMM(m)_ = 0.530; R^2^_LMM(c)_ = 0.724				
**AS**	(Intercept)	1, 14	716.62	<0.001
	**Mass**	**1, 11**	**6.81**	**0.02**
	Collected	1, 14	0.18	0.68
	**Treatment**	**1, 11**	**38.70**	**<0.001**
	Mass X Collected	1, 11	3.18	0.10
	Mass X Treatment	1, 11	0.50	0.49
	Collected X Treatment	1, 11	2.82	0.12
R^2^_LMM(m)_ = 0.530; R^2^_LMM(c)_ = 0.736				
**AR**	(Intercept)	1, 80	31153.48	<0.001
	*L*_T_	1, 80	1.33	0.25
	Collected	1, 14	0.25	0.63
	**Treatment**	**1, 80**	**9.26**	**<0.01**
	*L*_T_ X Collected	1, 80	0.13	0.26
	*L*_T_ X Treatment	1, 80	3.74	0.06
	Collected X Treatment	1, 80	1.09	0.30
R^2^_LMM(m)_ = 0.112; R^2^_LMM(c)_ = 0.355				

Fish mass (M), total length (*L*_T_), collection site (Collected), rearing treatment (Treatment), absolute maximum metabolic rate (MMR, mg O_2_ h^-1^), aerobic scope (AS, mg O_2_ h^-1^), pectoral fin aspect ratio (AR). R^2^_LMM(m)_ is the marginal R^2^ representing the variance explained by fixed factors in the model, and R^2^_LMM(c)_ is the conditional R^2^ representing the variance explained by fixed and random factors in the model. Factors in **bold** represent statistically significant effects (α = 0.05).

Wave-reared fish had greater blood Hct than calm-reared conspecifics (treatment F_1,15_ = 7.97, *P* = 0.01; site of origin: F_1,14_ = 0.46, *P* = 0.51; Table 1). There was no statistical difference in plasma cortisol levels between flow treatments (F_1,13_ = 0.48, *P* = 0.50) or site of origin (F_1,14_ = 0.24, *P* = 0.63).

**Fig 2:**
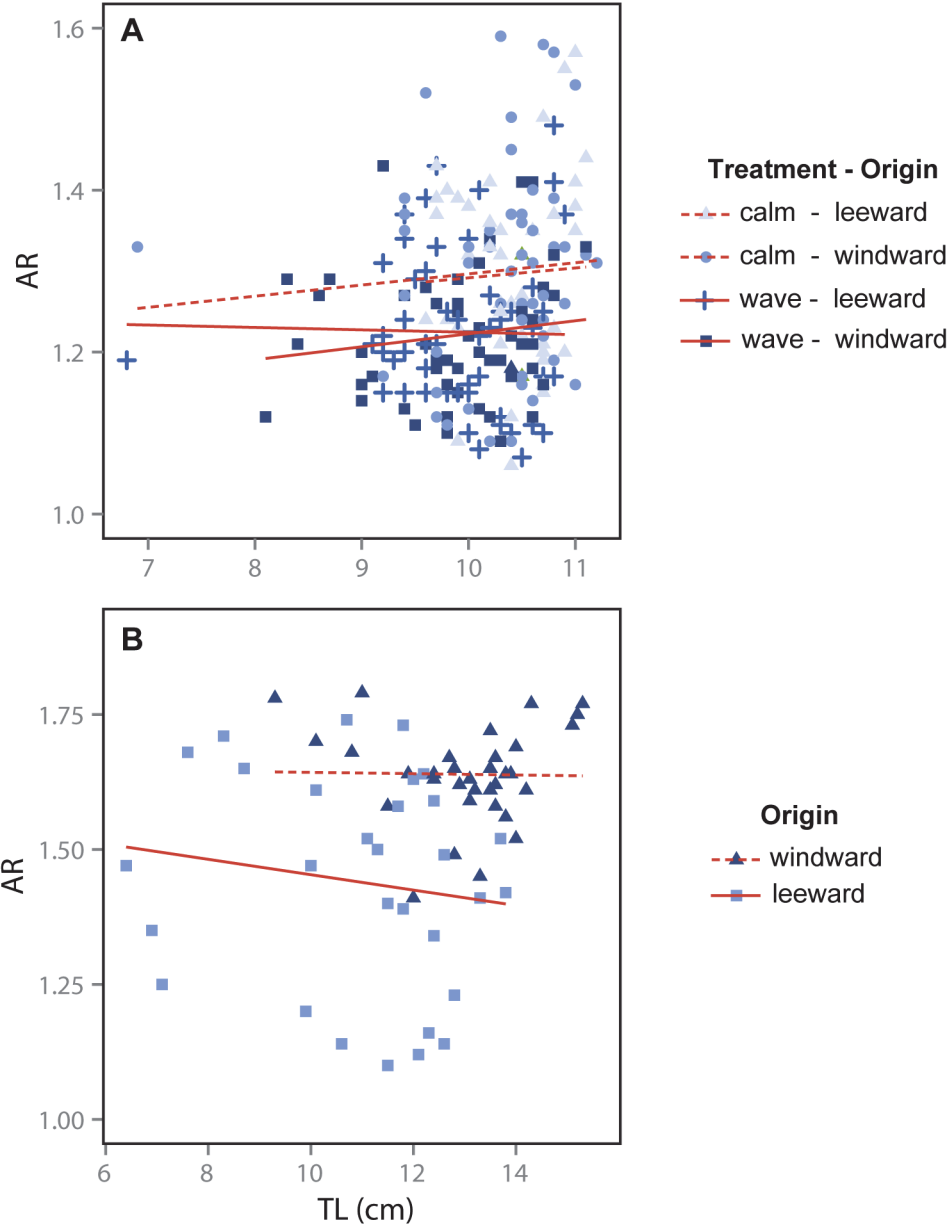
Pectoral fin aspect ratio (AR) in relation to fish total length (TL) in *Acanthochromis polyacanthus*. (A) Experimentally reared fish from different collection sites (leeward and windward) and rearing treatments (wave and calm; data from this experiment). (B) Wild fish from different collection sites (leeward vs. windward; data from Binning et al. 2014). The solid and dashed lines represent best fit linear regressions.

All broods displayed a range of pectoral fin shapes measured after four and eight months in the rearing treatment (AR range: 1.09–1.57 across all fish, S1 Table), and mean fin shape across treatments and collection sites was similar at both sampling times (Table 1). Fish *L*_T_ was not related to fin AR (Fig. 2A, Table 2). Although there was a significant effect of treatment on fin AR (*P* < 0.01), the difference in fin shape was small and in the opposite direction as predicted: fish from calm tanks had slightly more tapered fins (higher AR) than those from wave tanks (Fig. 2A, Tables 1 and 2). Collection site had no effect on fin AR (Table 2). These trends differ from what has been reported in wild fish. Wild adult *A*. *polyacanthus* collected from the same sites have higher pectoral fin AR in windward versus leeward areas around Lizard Island (Fig. 2B, Table 3; see [28]). Reared fish were smaller and had less tapered pectoral fins (lower AR) on average than wild fish (S1 Fig.). However, there was considerable overlap in both pectoral fin AR and fish *L*_T_ between the reared and wild individuals compared in this study (S1 Fig.).

**Table 3:** Comparison of swimming traits in wild and reared *Acanthochromis polyacanthus*.

	*U*_crit_	MMR	AS	AR
	(*L*_T_ s^-1^)	(mg O_2_ kg^-1^ h^-1^)	(mg O_2_ kg^-1^ h^-1^)	
**Collection site (wild fish)**				
windward: leeward	3.89: 2.99	452: 338	347: 220	1.64: 1.44
	Δ = 0.90	Δ = 114	Δ = 127	Δ = 0.20
	1.30: 1	1.34: 1	1.58: 1	1.14: 1
	*L*_T_ = 12.8: 13.1	M = 41.9: 41.0	M = 41.9: 41.0	
	N = 20	N = 20	N = 20	N = 63
**Collection site (reared fish)**				
windward: leeward	5.08: 5.07	489: 485	334: 330	1.25: 1.26
	Δ = 0.01	Δ = 4	Δ = 4	Δ = -0.01
	1: 1	1: 1	1.02: 1	1: 1.01
	*L*_T_ = 10.4: 10.4	M = 19.5: 18.4	M = 19.5: 18.4	
	N = 32	N = 32	N = 32	N = 101
**Treatment (reared fish)**				
wave: calm	5.42: 4.73	533: 442	381: 283	1.23: 1.28
	Δ = 0.69	Δ = 91	Δ = 98	Δ = - 0.05
	1.15: 1	1.21: 1	1.35: 1	1: 1.04
	*L*_T_ = 10.1: 10.7	M = 19.6: 18.3	M = 19.6: 18.3	
	N = 32	N = 32	N = 32	N = 101

Data for wild fish from Binning et al. 2014. Experimentally reared *A*. *polyacanthus* collected from windward and leeward sites around Lizard Island reared in either wave or calm tanks (this study). Absolute ratio, absolute difference (Δ), relative ratio, absolute fish sizes (***L***_T_ in cm or M in g) and total sample size (N) are presented for the following traits: relative critical swimming speed (*U*_crit_), maximum metabolic rate (MMR), aerobic scope (AS) and pectoral fin aspect ratio (AR) between windward and leeward collection sites or wave and calm treatments.

**Fig 3:**
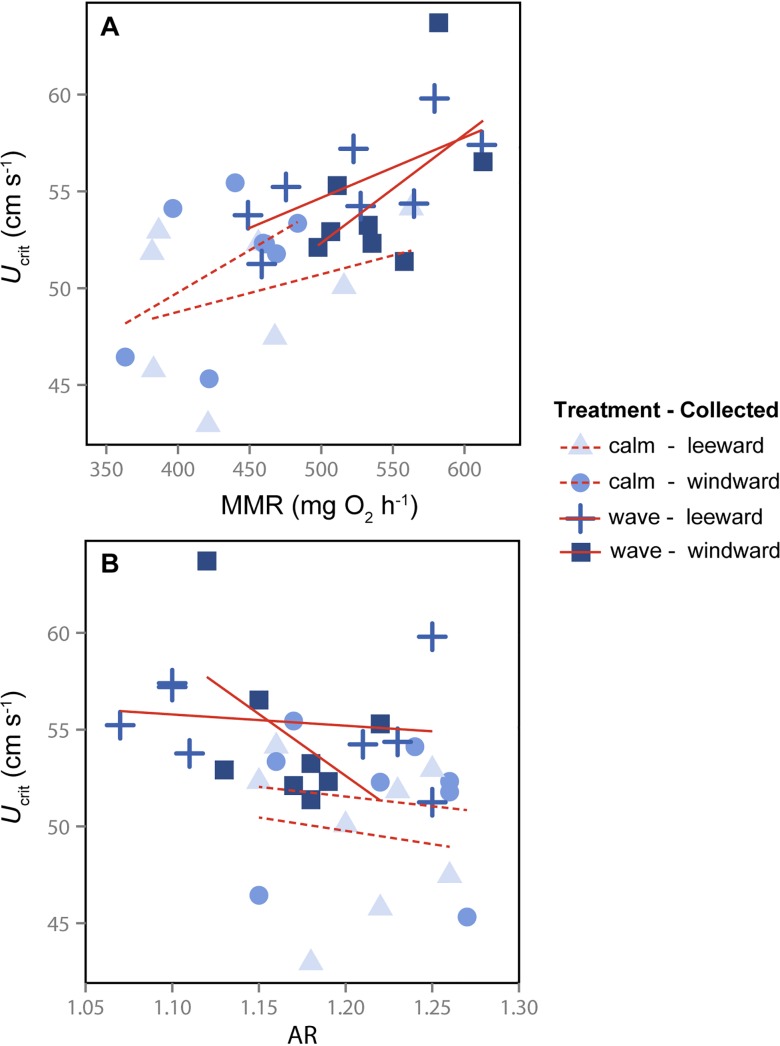
Relationships between swimming performance (*U*_crit_), metabolic rates and fin shape in *Acanthochromis polyacanthus*. (A) Absolute maximum metabolic rate (MMR) and (B) pectoral fin aspect ratio (AR) in individual fish from different collection sites (windward vs. leeward) and rearing treatments (wave vs. calm) are represented by shaded symbols. The solid and dashed lines represent the best fit linear regressions for wave and calm treatments, respectively.

Thirteen candidate models were within 10% of the strongest potential predictors of swimming performance. There was strong support for increased swimming performance (absolute critical swimming speed; *U*_crit_; cm s^-1^) with increased relative MMR. MMR appeared in all selected models, had the highest w_*ip*_ of all parameters, and had a positive parameter estimate that did not include 0 within the 95% CI (Fig. 3A; Table 4). Treatment also predicted swimming performance with fish from the wave treatment having a faster *U*_crit_ than fish from the calm treatment. The treatment effect was included in 7 of the 13 selected models, although the 95% CI overlapped 0. Treatment was about 2.5 times less likely than MMR to predict swimming performance, as indicated by the ratio of the sums of w_*ip*_ in models that included MMR compared to models that included treatment ([56]; Table 4).

**Table 4:** Predictors of swimming performance in *Acanthochromis polyacanthus*.

Predictor	Model Rank													β	SE	95% CI	w*ip*
	1	2	3	4	5	6	7	8	9	10	11	12	13				
**(Intercept)**	x	x	x	x	x	x	x	x	x	x	x	x	x	52.38	0.98	50.463 to 54.289	
**MMR**	x	x	x	x	x	x	x	x	x	x	x	x	x	2.44	0.79	0.903 to 3.970	1.00
**Treatment**			x	x			x		x		x	x	x	0.83	0.82	-0.769 to 2.433	0.40
**AR**		x					x	x		x			x	-0.20	0.23	-0.651 to 0.250	0.30
***L***_T_				x	x			x				x	x	0.21	0.25	-0.277 to 0.693	0.27
**Collected**						x				x	x			-0.05	0.18	-0.390 to 0.294	0.13
**MMR X Treatment**									x			x		0.07	0.13	-0.175 to 0.321	0.07
**K**	3	4	4	5	4	4	5	5	5	5	5	6	6				
**AICc**	170.98	172.05	172.11	172.89	173.11	173.53	174.03	174.28	174.63	174.74	174.83	175.00	175.25				
**ΔAICc**	0.00	1.07	1.13	1.91	2.13	2.55	3.05	3.30	3.65	3.76	3.85	4.03	4.27				
**R** ^2^_LMM(m)_	0.41	0.43	0.42	046	0.43	0.41	0.43	0.46	0.42	0.42	0.41	0.48	0.47				
**R** ^2^_LMM(c)_	0.50	0.50	0.50	0.55	0.54	0.51	0.49	0.55	0.48	0.51	0.50	0.58	0.56				
**w**_*im*_	0.23	0.14	0.13	0.09	0.08	0.07	0.05	0.04	0.04	0.04	0.03	0.03	0.03				

Variables included in each model are denoted with an “x.” Predictors are absolute maximum metabolic rate (MMR), rearing treatment (Treatment), pectoral fin aspect ratio (AR), fish total length (*L*_T_), collection site (Collected), and the interaction between absolute metabolic rate and rearing treatment (MMR X Treatment). The number of parameters (K) used in each model, the AIC_c_, the ΔAICc (AIC model _I_—AIC of best model), the marginal R^2^ (R^2^_GLMM(m)_; variance explained by the fixed factors), conditional R^2^ (R^2^_GLMM(c)_; variance explained by the fixed and random factors) and the w_*im*_ (normalized Akaike weights for each candidate model) are shown at the bottom of the table. Model averaged parameter estimates (β) unconditional standard errors (SE), 95% confidence intervals (95% CI) and the normalized Akaike weight for each predictor (w_*ip*_) are shown in the right hand columns. All models include a constant (Intercept).

Both pectoral fin AR and fish *L*_T_ were included in 5 of the 13 candidate models—their 95% CIs overlapped 0. Pectoral fin AR was 3.3 times and *L*_T_ 3.7 times less likely than MMR to predict differences in swimming performance among individuals (Fig. 3B; Table 4). Fish collection site and the interaction between MMR and *L*_T_ were included in only 3 and 2 of the candidate models, respectively, with CIs overlapping 0. They were 7.7 and 14.3 times less likely to predict differences in swimming performance than MMR.

The magnitude of relative differences in *U*_crit_, MMR and AS between windward and sheltered wild fish is similar to that of reared fish from calm and wave treatments, although the differences in these traits for wild fish are somewhat larger than differences for reared fish (Table 3).

## Discussion

Identifying morphological and/or physiological traits that contribute to performance differences is essential for understanding species distributions and patterns of selection across habitats. For mobile animals like most fishes, locomotor performance is critical for survival, and therefore fitness [7,24]. As a result, selection should act on traits enhancing locomotor performance in a given habitat, creating patterns of trait divergence both within and between species. Wild populations of *A*. *polyacanthus* on the Great Barrier Reef differ greatly in terms of their fin morphology, physiology and swimming performance [28,29]. However, a mechanistic explanation for this natural pattern of divergence as well as the relative importance of trait variation in driving performance differences was unknown. We show that critical swimming speed, one measure of prolonged swimming performance, was best predicted by maximum metabolic rate in *A*. *polyacanthus* (Figs. 1 and 3A). Our results also suggest that these physiological traits may enhance individual fitness in a given habitat, being plastically induced upon exposure to water flow during development: wave-reared fish tended to have higher MMR and AS than calm-reared fish regardless of the conditions experienced on their natal reef or brood identity. In addition, haematocrit, one component of blood oxygen carrying capacity, was 25% higher in wave-reared individuals (Table 1). These differences in Hct are not likely a stress response as blood cortisol levels were consistent across sites and water flow treatments. However, long term stress in animals can sometimes lead to a down-regulation in cortisol production, and therefore assessing cortisol levels may not be the best measure for assessing stress during long-term experiments such as this [58].

Many species of damselfishes are highly territorial, and rely on the shelters provided by the highly complex reef matrix for protection from predators [59]. Therefore, in fast-flowing water, damselfish must actively swim, sometimes at very high speeds, in order to maintain position on their territories. As a result, traits which increase swimming performance and oxygen carrying capacity are likely beneficial to *A*. *polyacanthus* in fast-flowing environments. We use critical swimming speed to assess the aerobic swimming performance of our fish as this is continually recognized as one of the best ecophysiological measurements of the swimming capabilities of planktivorous fishes such as *A*. *polyacanthus* [24,38]. We have not included estimates of anaerobic burst-performance in this study, but future studies could evaluate whether these traits trade-off with aerobic performance estimates in different flow environments.

Swimming performance in our fish was enhanced by physiological changes which occurred as a result of experimental exposure to high water flows. These results support findings from exercise training experiments in a variety of organisms: trained groups tend to have higher maximum metabolic rates and swimming performance than untrained groups [60–68]. Most of the research on swimming performance and exercise in poikilotherms is limited to a few species of commercially important temperate fishes [61]. Very little has been published on the exercise physiology of tropical marine fishes despite their growing presence in the aquaculture industry and susceptibility to environmental stressors (but see [48,61]). Furthermore, no studies have explored training effects in labriform swimmers, or effects due to irregular flow protocols with conditions similar to those experienced in shallow marine habitats. The training regime imposed by flows of varying speed used here may present a greater physiological challenge than constant-speed conditions typically used in exercise-training studies. Studies in temperate systems have shown that swimming costs are higher for fish in unsteady compared to steady flows for both BCF and labriform-swimming fishes [69,70]. Our results suggest that tropical marine fishes can enhance their swimming performance upon exposure to wave-like water flows by developing physiological traits that increase oxygen uptake and delivery. Studies exploring the response of adult coral reef fishes to exercise training are now needed to assess how labile these physiological adaptations are within a fishes’ lifespan.

The differences in the physiological traits measured between flow treatments in our rearing study broadly reflected those observed in wild adults ([28]; Table 3). The relative differences in *U*_crit_, MMR and AS between windward and leeward wild fish were similar to those of reared fish from calm and wave treatments (30, 34 and 58% higher performance respectively in wild, windward fish versus 15, 21 and 35% higher performance in wave-reared fish; Table 3). Conversely, relative values of *U*_crit_, MMR and AS between reared fish collected from windward and leeward habitats did not differ, suggesting that differences in performance traits were plastically induced by the rearing environment rather than the result of genetic differences between sites (Table 3). Our findings also suggest that the differences in swimming performance observed in wild adult populations of *A*. *polyacanthus* are likely explained by plastic physiological changes induced by water flow. Selection pressure on physiological traits promoting swimming performance are likely greater in the wild than in our controlled experiment, which may explain why the magnitude of differences in swimming performance between flow habitats was greater in wild individuals. This developmental plasticity may be an important mechanism promoting the survivorship of this, and potentially other species across water flow habitats, and should be considered in future research.

In labriform-swimming fishes, tapered pectoral fins promote high-speed swimming. This has been demonstrated both theoretically using drag-based models [20,22,71], and empirically across species with varying fin shapes [20,22,23,72]. Similar patterns relating fin shape and size to swimming speed have been observed in intraspecific studies [29,73]. However, fin shape was not a strong predictor of critical swimming speed in our study. These results are surprising as tapered pectoral fins are believed to be a fundamental trait necessary for the persistence of many coral reef fish species in wave-swept habitats [12,13,19]. One possible explanation is that our fish did not reach a large enough size before they were tested and measured, and therefore did not reach full trait expression. However, our reared fish overlapped in both fish *L*_T_ and fin AR with wild individuals which exhibit morphological divergence across water-flow habitats ([28]; S1 Fig.). Furthermore, fin AR does not vary with fish *L*_T_ in either reared or wild individuals (Fig. 2), suggesting that the size of reared fish would not impact the expression of fin shape. Our estimates of fin shape also did not change between four and eight months in the rearing treatment suggesting that overall fin shape does not vary markedly throughout ontogeny in this species (Table 1). It is also possible that by commencing the experiments using one month old fish, we missed a narrow developmental window wherein fin morphology can respond to environmental conditions. However, if fin morphology was developmentally fixed prior to experimental rearing, we might expect a strong site effect, which we did not observe (Table 2). A more realistic explanation for our results is that the links between organismal structure and function are complex, and fish form rarely influences performance in a simplistic cause-and-effect manner [3,25]. Rather, individual performance involves the complex integration of a number of traits involving slight differences in an organism’s physiology and biochemistry as well as morphology. Thus, the contribution of any single trait to overall performance may be small, but significant when integrated across a range of traits. Further investigation is necessary to understand the degree to which differences in morphology affect individual swimming performance when controlling for physiological traits such as metabolic rate.

Water flow induces plastic changes in the fin shape and length of several freshwater fish species [15,17,18]. However, we did not find evidence for phenotypic plasticity in determining the fin shape of *A*. *polyacanthus* (no treatment effect; Fig. 2), nor was there evidence that fin shape is strongly heritable (no site effect; Fig. 2): fish displayed a wide range of fin shapes within all broods sampled (S1 Table). There are several potential explanations for why within-brood variation in pectoral fin shape is high despite no apparent link with steady swimming performance. First, fishes use their fins for a wide variety of behaviours including manoeuvring through complex habitats, maintaining postural control and stability and engaging in territorial and foraging activities [35,74–76]. Gerstner [76] found that morphological differences among four species of coral reef fishes correlated with behavioural rather than performance differences. Fin shape may, therefore, be selected to optimize activities or behaviours other than prolonged swimming performance in these fish. Second, demersal fishes can exploit the physical structure of the reef to minimize the water flows experienced and expand their swimming potential via flow refuging [36]. As a result, morphology related to differences in habitat use may override the need for specialized prolonged swimming morphology in a given habitat. Our experimental tanks provided fish with shelters where they could avoid the flow and open-water, which reflected the presence of such refuges observed in natural reef habitats. Differences in shelter use and swimming behaviour by individuals within our tanks may, therefore, be more associated with fin shape differences than sustained swimming performance. Third, MPF swimmers have a decoupled locomotor system, whereby different body regions and appendages are used for kinematically different activities [77]. For example, most MPF-swimming fishes use the BCF swimming mode to generate forward thrust when swimming at high-speeds [74,77]. Thus, pectoral fins designed for high-speed swimming may not be under strong selection pressure if fish can use BCF locomotion during burst activities. A recent study found that patterns of fin shape polymorphism across flow environments were weaker in pomacentrids compared to other labriform-swimming families, likely due to the behavioural and physiological flexibility observed across this family [19]. Taken together, flexibility in habitat use and behaviour in this, and potentially other MPF-swimming species, may weaken selection on pectoral fin shape, especially if changes in physiological traits can occur rapidly and contribute significantly to overall swimming performance. Genetic drift may also be partially responsible for the high amounts of variation in fin shape observed, although it remains unclear why such consistent differences in fin shape would occur in wild populations along wave gradients across multiple spatial scales if there was no biomechanical advantage to this trait.

Studies in temperate systems suggest that water flow fluctuations can be energetically costly for fishes [69,70]. However, we found that a coral reef fish can adapt plastically to high flows with exposure to water motion throughout ontogeny. Wave reared individuals increased their swimming performance by 20–30% compared to individuals with limited experience in flowing water. Furthermore, our results conclusively attributed these swimming performance differences to physiological rather than morphological traits in this species. Do other widespread species show similar plasticity in physiological traits? What are the benefits of fin shape polymorphism in this system? These questions remain unanswered. Nevertheless, the ability of *A*. *polyacanthus* to adapt physiologically to changes in water flow suggests that these traits play an important role in influencing the distributions of individuals and species, and should be considered alongside morphological traits when evaluating form-function relationships and their effects on individual performance and fitness.

## Supporting Information

S1 Fig Comparison of pectoral fin aspect ratio and total length of reared and wild *Acanthochromis polyacanthus*. Frequency distributions of (A) pectoral fin aspect ratio and (B) fish total length (*L*_T_) in fish reared in experimental tanks for 8 months (this experiment, light blue distributions, N = 100) and from wild individuals (data from Binning et al. 2014; dark blue distributions, N = 63). Distributions have been scaled to account for differences in sample size between the two populations.
(PDF)

S1 Table Pectoral fin shape metrics for 16 *Acanthochromis polyacanthus* broods. Broods were collected from leeward (sheltered) and windward (exposed) sites surrounding Lizard Island after eight months of experimental rearing. The number of fish fins measured (N), range of fin aspect ratios (Min-Max AR) and the mean aspect ratio with standard deviation (Mean AR ± SD) are presented.
(PDF)
