# Supplementary material for: Physiological Plasticity to Water Flow Habitat in the Damselfish, Acanthochromis polyacanthus: Linking Phenotype to Performance
Source: PLoS One. 2015 Mar 25;10(3):e0121983. doi: 10.1371/journal.pone.0121983 (PMC4373956; doi:10.1371/journal.pone.0121983)

S1 Figure Comparison of pectoral fin aspect ratio and total length of reared and wild *Acanthochromis polyacanthus*. Frequency distributions of (A) pectoral fin aspect ratio and (B) fish total length (L_T) in fish reared in experimental tanks for 8 months (this experiment, light blue distributions, N = 186) and from wild individuals (data from Binning et al. 2014; dark blue distributions, N = 63). Distributions have been scaled to account for differences in sample size between the two populations.

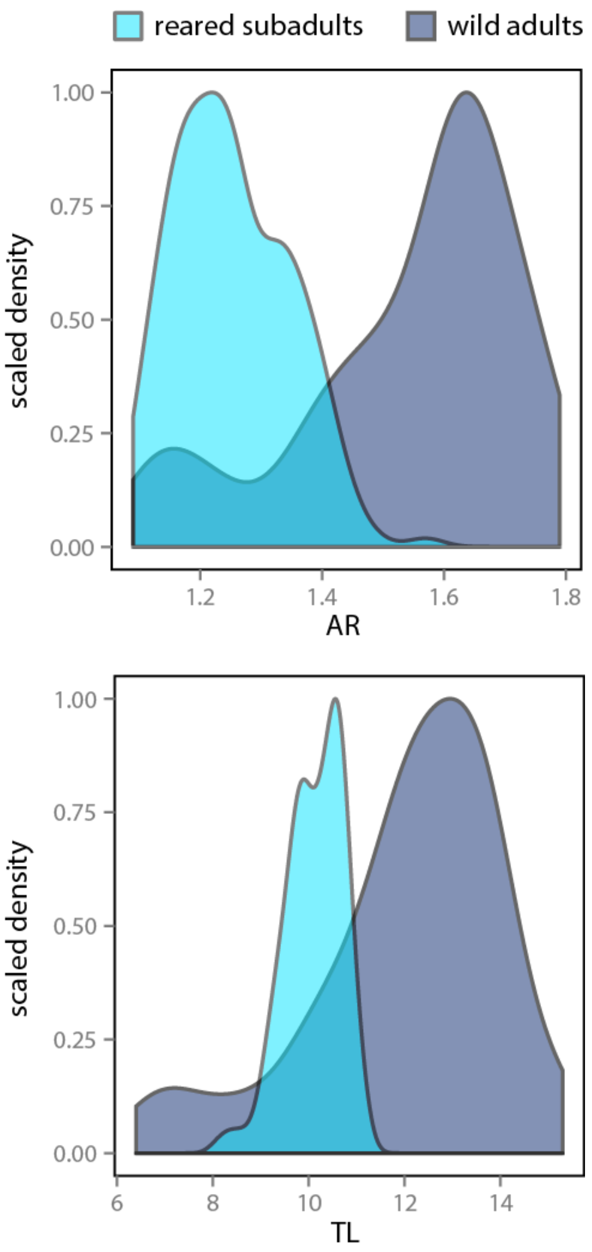

Supplement: S1 Fig — Frequency distributions of (A) pectoral fin aspect ratio and (B) fish total length (L T) in fish reared in experimental tanks for 8 months (this experiment, light blue distributions, N = 100) and from wild individuals (data from Binning et al. 2014; dark blue distributions, N = 63). Distributions have been scaled to account for differences in sample size between the two populations. (PDF) [file pone.0121983.s001.pdf]
