# Supplementary material for: Physiological Plasticity to Water Flow Habitat in the Damselfish, Acanthochromis polyacanthus: Linking Phenotype to Performance
Source: PLoS One. 2015 Mar 25;10(3):e0121983. doi: 10.1371/journal.pone.0121983 (PMC4373956; doi:10.1371/journal.pone.0121983)
Supplement: S1 Table — Broods were collected from leeward (sheltered) and windward (exposed) sites surrounding Lizard Island after eight months of experimental rearing. The number of fish fins measured (N), range of fin aspect ratios (Min-Max AR) and the mean aspect ratio with standard deviation (Mean AR ± SD) are presented. (PDF) [file pone.0121983.s002.pdf]

S1 Table Pectoral fin shape metrics for 16 *Acanthochromis polyacanthus* broods. Broods were collected from leeward (sheltered) and windward (exposed) sites surrounding Lizard Island after eight months of experimental rearing. The number of fish fins measured (N), range of fin aspect ratios (Min-Max AR) and the mean aspect ratio with standard deviation (Mean AR ± SD) are presented.

Brood	N	Min-Max AR	Mean AR ± SD
**Leeward**			
1	14	1.11 - 1.43	1.25 ± 0.10
2	9	1.12 - 1.48	1.24 ± 0.13
3	10	1.17 - 1.37	1.28 ± 0.07
4	15	1.09 - 1.57	1.26 ± 0.13
5	11	1.11 - 1.40	1.26 ± 0.11
6	12	1.18 - 1.43	1.31 ± 0.08
7	10	1.15 - 1.32	1.22 ± 0.06
8	9	1.10 - 1.39	1.25 ± 0.09
**Windward**			
9	14	1.10 - 1.37	1.23 ± 0.08
10	10	1.14 - 1.36	1.24 ± 0.08
11	12	1.12 - 1.43	1.23 ± 0.09
12	14	1.11 - 1.21	1.25 ± 0.10
13	16	1.13 - 1.45	1.26 ± 0.10
14	7	1.12 - 1.33	1.23 ± 0.09
15	9	1.09 - 1.33	1.22 ± 0.08
16	14	1.12 - 1.41	1.28 ± 0.08
**All fish**	186	1.09 – 1.57	1.25 ± 0.09
